# RETRACTED: Yang et al. An Adaptive Hierarchical Network Model for Studying the Structure of Economic Network. *Entropy* 2022, *24*, 702

**DOI:** 10.3390/e26010006

**Published:** 2023-12-20

**Authors:** Xiaoteng Yang, Zhenqiang Wu, Shumaila Javaid

**Affiliations:** 1 Key Laboratory of Modern Teaching Technology, Ministry of Education, Xi’an 710062, China; yxt@snnu.edu.cn; 2 School of Computer Science, Shaanxi Normal University, Xi’an 710062, China; 3 Department of Control Science and Engineering, College of Electronics and Information Engineering, Tongji University, Shanghai 201804, China; shumaila@tongji.edu.cn; 4 Frontiers Science Center for Intelligent Autonomous Systems, Shanghai 201210, China

This journal retracts the article, ‘An Adaptive Hierarchical Network Model for Studying the Structure of Economic Network’ [1]. 

Following publication, concerns were raised to the Editorial Office relating to an overlap with a previously published article [2] authored by a different authorship group without appropriate citation or acknowledgment.

Adhering to our complaints procedure, an investigation was conducted by the Editorial Office and Editorial Board, which confirmed a high level of overlap that included a number of identical figures and theorems, as well as analysis of the ideas. Therefore, the Editorial office and the Editorial Board decided to retract this article. 

This retraction was approved by the Editor-in-Chief of the journal *Entropy*.

The authors agreed to this retraction.

## References

[1] Yang X., Wu Z., Javaid S. (2022). RETRACTED: An Adaptive Hierarchical Network Model for Studying the Structure of Economic Network. Entropy.

[2] Kawakatsu M., Chodrow P.S., Eikmeier N., Larremore D.B. (2021). Emergence of hierarchy in networked endorsement dynamics. Proc. Natl. Acad. Sci. USA.

